# Uterine Segmentation and Volume Measurement in Uterine Fibroid Patients’ MRI Using Fuzzy C-Mean Algorithm and Morphological Operations

**DOI:** 10.5812/kmp.iranjradiol.17351065.3142

**Published:** 2011-11-25

**Authors:** Alireza Fallahi, Mohammad Pooyan, Hossein Ghanaati, Mohammad Ali Oghabian, Hassan Khotanlou, Madjid Shakiba, Amir Hossein Jalali, Kavous Firouznia

**Affiliations:** 1 Department of Biomedical Engineering, Hamedan University of Technology, Hamedan, Iran; 2 Department of Biomedical Engineering, Shahed University, Tehran, Iran; 3 Department of Radiology, Advanced Diagnostic and Interventional Radiology Research Center (ADIR), Imam Khomeini Hospital, Tehran University of Medical Sciences, Tehran, Iran; 4 Department of Medical Physics, Research Center for Science and Technology in Medicine, Tehran University of Medical Sciences, Tehran, Iran; 5 Department of Computer Engineering, Bu Alisina University, Hamedan, Iran

**Keywords:** Uterine Fibroids, Magnetic Resonance Imaging, Patients

## Abstract

**Background:**

Uterine fibroids are common benign tumors of the female pelvis. Uterine artery embolization (UAE) is an effective treatment of symptomatic uterine fibroids by shrinkage of the size of these tumors. Segmentation of the uterine region is essential for an accurate treatment strategy.

**Objectives:**

In this paper, we will introduce a new method for uterine segmentation in T1W and enhanced T1W magnetic resonance (MR) images in a group of fibroid patients candidated for UAE in order to make a reliable tool for uterine volumetry.

**Patients and Methods:**

Uterine was initially segmented using Fuzzy C-Mean (FCM) method in T1W-enhanced images and some morphological operations were then applied to refine the initial segmentation. Finally redundant parts were removed by masking the segmented region in T1W-enhanced image over the registered T1W image and using histogram thresholding. This method was evaluated using a dataset with ten patients’ images (sagittal, axial and coronal views).

**Results:**

We compared manually segmented images with the output of our system and obtained a mean similarity of 80%, mean sensitivity of 75.32% and a mean specificity of 89.5%. The Pearson correlation coefficient between the areas measured by the manual method and the automated method was 0.99.

**Conclusions:**

The quantitative results illustrate good performance of this method. By uterine segmentation, fibroids in the uterine may be segmented and their properties may be analyzed.

## 1. Background

Uterine fibroids are the most common benign tumors of the female pelvis affecting 20-50% of the women in the world [1]. Different methods, such as magnetic resonance imaging (MRI) are used in the diagnosis of this disease. In fact, MR imaging can be very useful in the diagnosis of the fibroids and assessing their treatment process and follow-up of the patients [2]. There are a few treatment options for the disease; one of which is uterine artery embolization (UAE). In this method, sacrificing the vascular bed of the uterine arteries results in fibroid shrinkage; subsequently, leading to decrease of the volume of the uterus and fibroid; therefore diminishing the patients’ symptoms. According to the nature of this procedure, measurement of uterine volume is very important in the follow-up of the treatment response. In this case, uterine volume measurement is one of the important tasks usually performed manually, which is difficult and time consuming. One good solution could be an automated method for measurement of the uterine volume on MRI images. In fact, by automated and semi-automated segmentation techniques, we can assist physicians to have a fast and more reproducible volumetry in comparison to common manual volumetry techniques. Automatic uterine and fibroid segmentation in MRI is a new research domain and little work has been reported in the literature. In one study which was performed by Clark et al. [3] the authors used base and fuzzy classification method for tumor segmentation and have proposed automatic segmentation by using the statistical classification method and atlas registration was done by Kaus et al. [4] for small brain tumors. The expectation maximization (EM) algorithm and atlas prior information were used in 2002 by Moon et al. [5]. A support vector machine (SVM) classification method, which needs a learning process and some user interactions, was recently applied [6][7]. There are a few papers published on automated fibroid segmentation. Guyon et al. [8] used geodesic active contours and a fast marching level set method combined with rigid landmark registration in order to track the tumor size variation over the time. Later on, Jianhua et al. [9] applied the fast marching level set method for initial segmentation, then Laplacian level set was used to refine the segmentation result. To the best of our knowledge, there are no papers on uterine segmentation.

## 2. Objectives

In this paper, we applied fuzzy C-means method and some morphological operations to segment the uterus on T1W and enhanced-T1W MR images. At the end, we evaluated the accuracy of the method using relevant indices.

## 3. Patients and Methods

### 3.1. Patient Selection

Ten patients with symptomatic fibroids and candidates for UAE were enrolled in the study. Among them, totally 120 MRI images (slices) were acquired. All the patients were imaged on a 1.5T GE MRI machine, using standard clinical imaging protocols, T1W and enhanced-T1W protocols. Each MR imaging was performed in an in-plane resolution of 0.78 mm and a slice thickness of 7 mm. In each patient, the segmentation method was applied in the sagittal, axial and coronal views. For each patient, the final volume was obtained by averaging three view volumes calculated in both automated and manually segmented images.

### 3.2. Data Evaluation

All images were reviewed by an expert radiologist and the borders of the uterus were highlighted on the digital images using the MIPAV program. Then, we ran our semi-automated method on the images and obtained the results. We then calculated the sectional areas of the uterus determined by each method. We used six measures; namely, the similarity index, Jaccard index, sensitivity, specificity, Hausdorff distance and the mean distance to evaluate the results which are mentioned below.

• Similarity index: *S_i_* = *2N_T_P__* /* N_M_ + N_A_* × 100%

Where M, denotes the manually segmented area; A, denotes the automated segmented area; *N_T_P__*, is the number of true positive voxels; * N_M_*, is the cardinality of M and *N_A_*, is the cardinality of A.

• Jaccard index: *J_i_* = *N_T_P__* / *N_M_* + *N_A_* + *N_T_P__* × 100%

• Ratio of correct detection (sensitivity):

*T_P_* = *N_T_P__* / *N_M_* × 100%

• Specificity: S_E_ = 100 - F_p_ where *F_P_* = *N_F_P__* / *N_A_* × 100% 

Where *N_F_P__* denotes the number of false positive voxels.

• Hausdorff distance between A and M (HD): defined as

*D_H_*= *max [h (M, A) h(A, M)]*

where *h(M, A)* = *max _mɛM_ min _aɛA _d(m, A) and d(m, a)*

Denotes the Euclidean distance between *m* and *a (m *and *a* are points of M and A, respectively).

• Mean distance *(MD)*: Average distance between the surfaces of *M* and *A*

The *Tp* value indicates how much of the actual tumor has been correctly detected, while *Fp* indicates how much of the detected tumor is wrong. The similarity index *S* is more sensitive to differences in location. For example, if region *A* completely includes region *M*, while *M* is one half of *A*, then the *Tp* value is 100% and the *S* value is 67%. Since usually most errors are located at the boundary of the segmented regions, small regions will have smaller *S* and *Tp* values than large regions. Therefore, we also use the average distance and the Hausdorff distance that do not depend on the region size. However, this measure is extremely sensitive to outliers and may not reflect the overall degree of matching.

### 3.3. Initial Uterine Segmentation With FCM Algorithm

Here, our aim was to propose an automatic method for initial segmentation of the uterine. The proposed method is based on an unsupervised classification (clustering), because this type of classification does not require user interaction, learning or prior models; hence, it may be automated easily.

Clustering is the partitioning of unlabeled data set

X = {x1, x2, ..., xn} ⊂ R^p^ into 1 < c < n classes, by assigning labels to the vectors in X. A c-partition of X is a set of (cn) values u_ik_ that can be represented as a (c × n) matrix U = [u_ik_] [10]. The value u_ik_ denotes the membership degree of sample x_k_ to class i.

One of the most widely used clustering methods is the FCM algorithm [11]. The FCM algorithm assigns memberships to x_k_, which are related to the relative distance of x_k_ from the c points prototypes V = {v_i_} that are class centers in the FCM.

**Figure 1:**
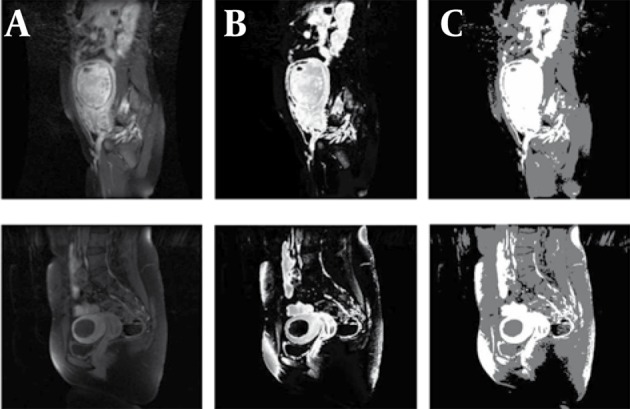
A, Sagittal Views of Enhanced-T1 MR Images for Two Samples; B, Result of Applying FCM Algorithm; C, Three Class Segmented Result

Because of texture properties in the enhanced-T1 MR image, we chose c = 3 and m = 2. As the uterus is enhanced in T1-enhanced images, their pixels appear in third class. Figure 1 shows the initial segmentation of two different cases using the FCM algorithm.

### 3.4. Morphological Operations

For many biomedical image analysis tasks, one usually focuses on a particular structure contained in a given image. In this research, the segmentation results are intended for the study of the uterus. Ideally, upon FCM clustering, the cluster corresponding to the brightest region would represent the uterus. The representation may be obtained by converting the clustering result into a binary image, in which each pixel is labeled as either belonging or not belonging to the desired region while representing the uterus. However, the region so obtained may not be fully connected and may contain other region pixels (such as colon due to the anatomical ambiguities). Due to the spatial smoothness that may be applied through these operations, basic morphological operations are doubtful [12][13]. Besides, for the correction of the definite large miss-clustered sites that are presented in the FCM segmentation results, the morphological operations may be used.

**Figure 2:**
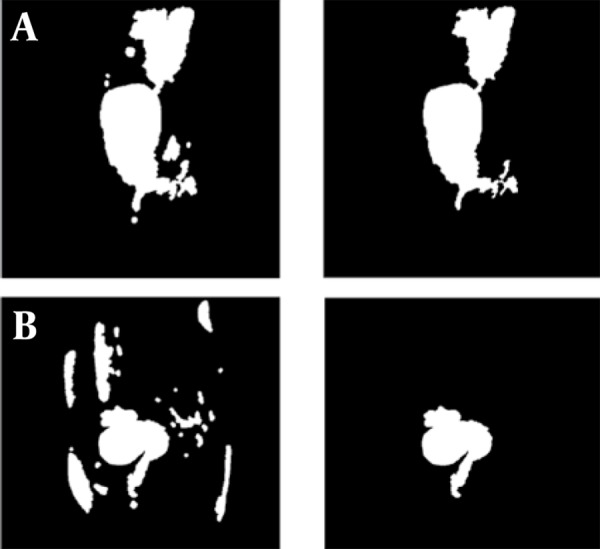
A and B, Result of Applying Morphological Operation

For clarification of anatomical ambiguities, two different morphological operations are used for binary images. As some holes will appear after the FCM clustering in infarcted fibroids, we have performed a filling algorithm to fill these holes. Then the opening was applied for the image to remove small isolated regions and to disconnect poor connectivites which do not belong to the region. The largest site in each cross section located in the center of the image is chosen as the candidate region belonging to the uterus (Figure 2). The anatomical ambiguity due to the lack of clear boundary between the uterus and the colon in the enhanced-T1 image would still be present after the initial morphological operations. Elimination of this ambiguity needs to utilize the registered T1 image which is described in the next section.

### 3.5. Segmentation Refinement by T1 Image

**Figure 3:**
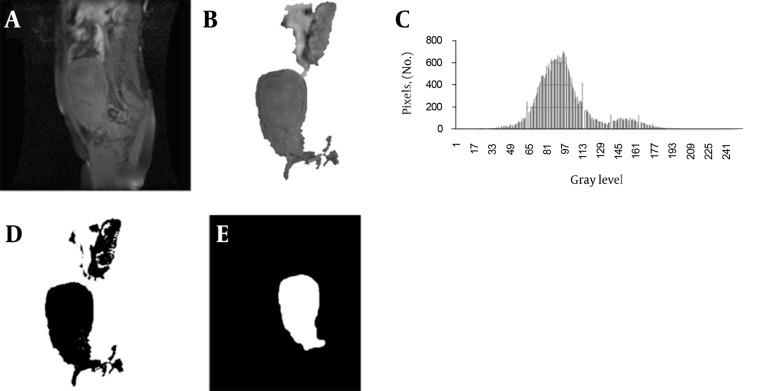
A, Registered T1 Image; B, Result of Applying Segmented Region as a Mask on T1 Image; C, Histogram of the Region; D, Result of Applying Threshold; E, Final Segmented Region

**Figure 4:**
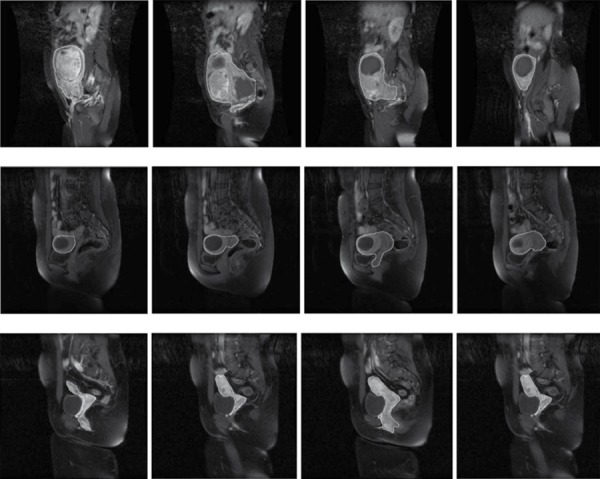
A 38-Year-Old Woman With Uterine Fibroid, Results of Automatic Segmentation of the Sagittal Images of MRI by Proposed Method

The necessity of further identification of the region-of-interest (ROI) is due to resolve the remained segmentation problems. For the analogous signal intensity, we classified some parts of the colon which was connected to the upper part of the uterus as the uterus. As we mentioned previously, such an ambiguity cannot be differentiated solely by the intensity and by using a threshold in the image we can eliminate these regions. In order to extract the segmented region from T1 image we should register T1 image to the enhanced-T1 image and for registration of the images, we used MIPAV software package. We chose the affine twelve degree transformation, trilinear interpolation and correlation ratio similarity function. The registration tools of this package use a local-global optimization method [14]. After registration, the segmented result was applied as a mask on the T1 image and this new image contains the uterus and the colon, in which we may use a threshold to eliminate (using a histogram). As the colon regions are brighter than the uterus, we select the start of the last peak as the threshold value. By using this threshold, other redundant parts are eliminated and the uterus may be obtained. In some cases, after these corrections, redundant parts that have the same signal intensity in T1 and enhanced-T1 images still remained and we eliminated these parts by applying opening followed by closing with a structure element that depends on the size of the region. Refinement of the segmentation results using the T1 image is shown in Figure 3. The final result of automatic segmentation by proposed method is shown in Figure 4.

## 4. Results

Quantitative results in 10 cases which were gathered by comparing the automatic segmentations with the available manual segmentations are showed in Table 1.

**Table 1:** Evaluation of the Segmentation Results of Images for Which a Manual Segmentation was Available

**Patient**	** Volume, mm^3^**		** Volume Metric, %**				**Distance Metric, mm**	
	**Manual**	**Automated**	**SI a**	**JI **a****	**SE **a****	**SP **a****	**HD **a****	**MD **a****
1	272960	293720	84.78	75.92	78.53	95.41	10.83	4.40
2	458251	482003	88.01	79.80	84.57	93.28	13.50	2.22
3	56390	56849	74.14	60.58	68.38	83.29	13.90	4.47
4	37477	44176	80.25	68.60	74.88	87.27	11.33	8.10
5	76915	78968	78.74	67.23	74.46	87.94	13.62	4.52
6	123031	119451	74.48	65.93	72.01	89.98	16.50	6.30
7	35950	41987	74.43	60.55	66.78	91.92	14.10	5.30
8	29907	33234	80.80	70.35	81.34	83.65	6.39	0.96
9	42048	60423	80.29	67.55	72.64	92.52	11.50	3.36
10	55026	64719	79.51	67.39	79.57	89.82	13.30	2.53
Mean ± SD	118795.5 ± 0	127553 ± 0	79.54 ± 4.51	68.39 ± 5.98	75.32 ± 5.67	89.51 ± 4.01	12.49 ± 4.01	4.21 ± 2.70

^a^ Abbreviations: HD, Hausdorff distance; JI, Jaccard index; MD, Mean distance; SE, Sensitivity; SI, Similarity index; SP, Specificity

The uterine size varies from 29907 mm^3^ to 458251 mm^3^. The similarity index varies from 74.14% to 88% with a mean of 80%. The Jaccard index varies from 60.55% to 79.92% with a mean of 68.39% and the correct detection ratio between 66.78% and 84.57% with a mean of 75.32%. Specificity ratio ranges between 83.29% and 95.41% with an average of 89.51% which shows a good accuracy of segmentation. The Hausdorff distance, which is the maximum distance and therefore a particularly precise evaluation, varies from 6.3 mm to 16.5 mm with a mean of 12.4 mm. The mean value of the average distance is 4.21 mm that is approximately equal to the the voxel size, and constitutes good result. As seen in Table 1, the best result is related to the largest uterine and worst result is related to the third and eighth cases that have smaller volumes.

The mean uterus area in the manual method was 4654 ± 3837.9 mm^2^ and in the semi-automated method was 4130.9 ± 3526.1 mm^2^ (P < 0.001). The mean percent of area difference between the two methods was 11.1% ± 17.4 [95.2% - 45.4%]. The Pearson correlation coefficient of the two methods was 0.976 (P < 0.001). In the linear regression model for estimating the manual area from semi automated value, the model R^2^ was 0.95 (P < 0.001) and the only independent variable (semi automated area) was statistically significant in the model.

The derived model was as follows:

S_Man_ = 1.061 × S_Aut_ + 269.094

**Figure 5:**
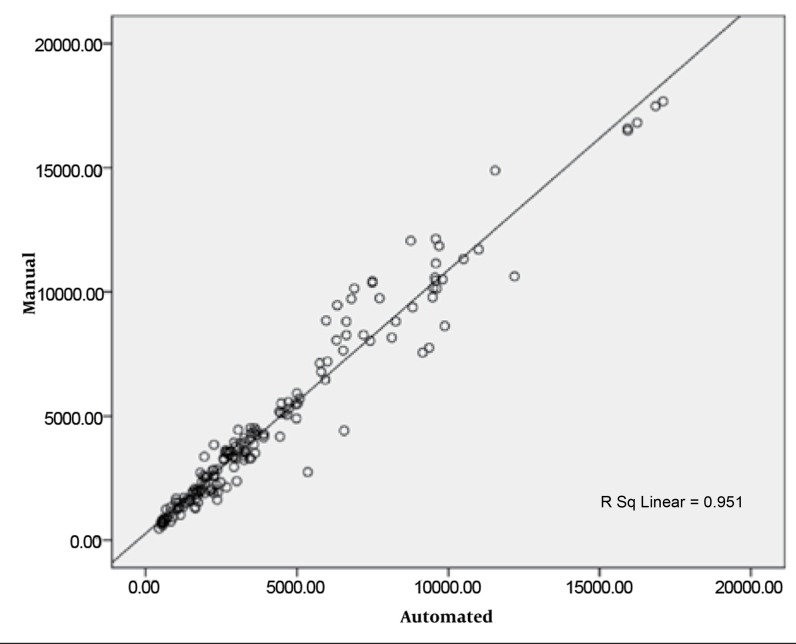
Scatter Plot Between Manual and Proposed Methods

The scatter plot between these two methods has been shown in Figure 5.

**Figure 6:**
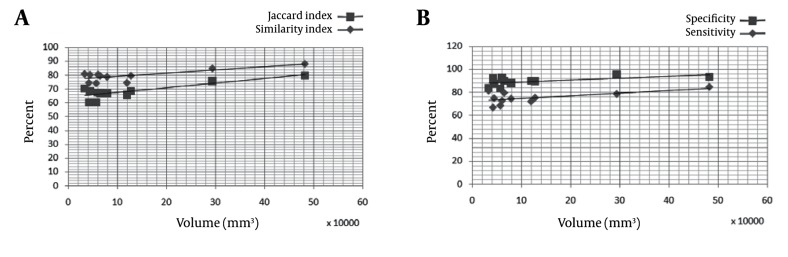
Representation Validation Parameters for Each Patient Versus Uterine Volume

**Figure 7:**
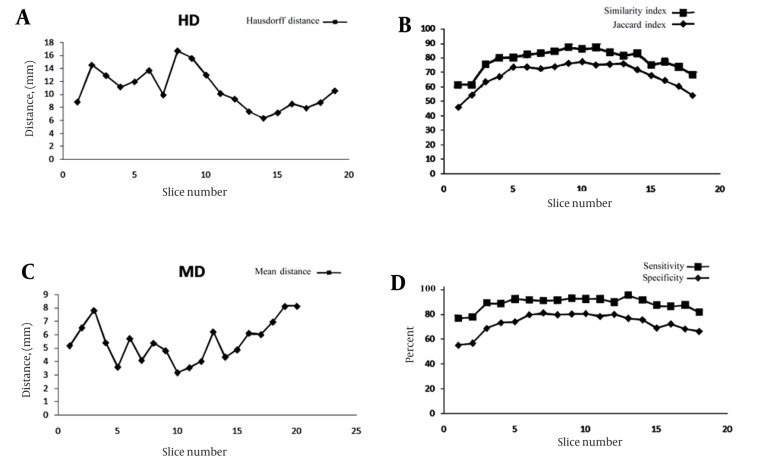
Validation Parameters in Each Slice for All Patients

Figure 6 shows volume metrics for each patent versus the size of the uterine. Figure 7 shows volume metric and distance metric in each slice.

## 5. Discussion

Uterine fibroids are the most benign tumors of the female pelvis. UAE is an effective treatment in patients with symptomatic uterine fibroids. Uterus segmentation and volume measurement has an important role in the extraction of fibroids characteristics, fibroids segmentation and follow-up of the patient’s condition. As the uterine enhanced in enhanced-T1W MR images and its intensity differed from other tissues, we used the FCM algorithm for its initial segmentation. Then T1W images were used for refine segmentation and eliminating redundant parts. FCM is a suitable algorithm for this tissue. Evaluation results showed a reliable overlap between automated and manual segmentations. However, some drawbacks may be considered. In some cases, infarcted fibroids that have low signal intensity create unconnected regions in uterine boundaries. In these cases, more processing must be used for reconstructing the shape of the uterine. Another problem occurred when some tissues had a similar intensity with the uterine in the enhanced-T1W and T1W images. In this case, shape features may be used for eliminating redundant parts.

As seen in (Figures 7A and B), Hausdorff distance has a higher value and mean distance has a lower value in the middle slices that have the largest volume. This suggests that in large volume objects, the outlier has a high value and causes high Hausdorff distance. For the mean distance, we calculated the mean distance between manual and automatic segmentation results that causes the distance normalized with object volume and has a lower value. Figure 7 shows volume metric in each slice. Because of normalizing this factor by object volume, this metric has a larger value in the middle slices and a lower value in the beginning and ending slices. In Figure 6, we can see that the volume metrics have larger values in patients with larger uterine volumes and lower values in patients with smaller uterine volumes. This result is similar to the information demonstrated in Figures 7C and D. A few works have been reported [8][9] in this area that have focused on uterine fibroid segmentation. However, no work is reported on uterine segmentation. In Yao study [9], the authors obtained 84.6% of sensitivity and 84.3% of specificity.

In conclusion, this paper proposed an automatic method for segmentation of the uterus in MR images. By using fuzzy C-means algorithm and some morphological operations, T1 images have firstly been segmented. The resulted image was refined by the registered T1-enhanced image and histogram processing. Finally, some morphological operations were applied as a post processing. The quantitative results showed a good performance of this method. By segmentation of uterine fibroids, in the future we can analyze their properties which have an essential role in diagnosis and treatment of uterine fibroids.
